# Matching Behavior as a Tradeoff Between Reward Maximization and Demands on Neural Computation

**DOI:** 10.12688/f1000research.6574.2

**Published:** 2015-10-02

**Authors:** Jan Kubanek, Lawrence H. Snyder

**Affiliations:** 1 Department of Anatomy and Neurobiology, Washington University School of Medicine, St. Louis, MO, 63110, USA; 2 Department of Biomedical Engineering, Washington University in St. Louis, St. Louis, MO, 63130, USA

**Keywords:** matching law, reward magnitude, reinforcement learning, value, choice, neurons

## Abstract

When faced with a choice, humans and animals commonly distribute their behavior in proportion to the frequency of payoff of each option. Such behavior is referred to as matching and has been captured by the matching law. However, matching is not a general law of economic choice. Matching in its strict sense seems to be specifically observed in tasks whose properties make matching an optimal or a near-optimal strategy. We engaged monkeys in a foraging task in which matching was not the optimal strategy. Over-matching the proportions of the mean offered reward magnitudes would yield more reward than matching, yet, surprisingly, the animals almost exactly matched them. To gain insight into this phenomenon, we modeled the animals' decision-making using a mechanistic model. The model accounted for the animals' macroscopic and microscopic choice behavior. When the models' three parameters were not constrained to mimic the monkeys' behavior, the model over-matched the reward proportions and in doing so, harvested substantially more reward than the monkeys. This optimized model revealed a marked bottleneck in the monkeys' choice function that compares the value of the two options. The model featured a very steep value comparison function relative to that of the monkeys. The steepness of the value comparison function had a profound effect on the earned reward and on the level of matching. We implemented this value comparison function through responses of simulated biological neurons. We found that due to the presence of neural noise, steepening the value comparison requires an exponential increase in the number of value-coding neurons. Matching may be a compromise between harvesting satisfactory reward and the high demands placed by neural noise on optimal neural computation.

## Introduction

People and animals must make choices. It has been often reported that organisms distribute the frequency of their choices according to the relative rate of reinforcement they obtain from each choice ^1– 4^. The match between the behavioral and reinforcement distributions in a two-option task has been described by the matching law:

$$\frac{B_{x}}{B_{x}+B_{y}}=\frac{R_{x}}{R_{x}+R_{y}},\;(1)$$

where *B _x_* and *B _y_* are the rates of behavior allocated at options *x* and *y*, and *R _x_* and *R _y_* are the corresponding rates of reinforcement obtained from these options ^1, 4^.

This elegant relationship has provoked much discussion and research across multiple fields ^3, 5– 9^. Although matching has been observed in many environments, including real-life settings ^10– 12^, there are important constraints on the conditions in which matching is observed.

First, matching behavior in the above form is consistently observed specifically in tasks that use or can be characterized by concurrent variable interval (VI-VI) schedules of reinforcement ^13, 14^. In such tasks, a reward is scheduled at an option after a certain interval and remains available until it is harvested. In these VI-VI paradigms, it is a sensible strategy for the decision-maker to occasionally select even the much poorer of the two options, since after a long enough interval, the animal can be sure that a reward will appear at that option ^6^. The VI-VI paradigms make matching an optimal or near-optimal strategy. In such tasks, matching follows from the maximization of reward at either the molecular (maximizing reward at each element of time) ^15– 17^ or molar (maximizing reward over the course of the experiment) ^13, 18, 19^ levels.

Second, matching is adversely affected by the animals’ tendency to often switch from one option to the other (e.g., ^1^, Figure 4). This frequent switching brings the proportion of choices of the two options closer to 50:50, which results in “under-matching” of the reward proportions. Such under-matching, as well as other deviations from the matching law, can be captured using generalized forms of the matching law ^20– 22^. Nonetheless, these generalizations come at the expense of freely adjustable parameters, thus diminishing the beauty of the matching equation. To discourage this behavioral tendency, researchers often punish the animals’ frequent switching by incorporating change-over delays (COD) ^1, 23– 25^. In a change-over delay paradigm, when an animal changes a choice, no reward is scheduled until a certain amount of time following the change. This effectively discourages frequent switching, and animals then often exhibit the matching behavior captured by Equation 1.

We engaged monkeys in a reward-magnitude-based foraging task that featured neither a VI-VI schedule nor a change-over delay. In our task, animals chose an option based on the magnitude (amount) of fluid reward expected for each option. The mean magnitude ratios for the two options, 3:1, and 1.5:1, changed often and unpredictably. Intriguingly, we observed a nearly exact matching of the magnitude ratios.

The finding that matching behavior is observed in a task that does not impose it provides important insights into the nature of matching behavior. To shed light on the mechanism, we described the animals’ behavior using a mechanistic model. The model faithfully captured the monkeys’ molar and molecular behavior. We show which components of the model are important in mediating matching. We then implement the critical component by populations of spiking neurons. The mechanistic modeling revealed a bottleneck in the animals’ ability to compare the values of the two options. The additional neuronal implementation suggested that this bottleneck could be due to noise in the representation of value by the neuronal populations.

## Methods

### Subjects

Two adult male rhesus monkeys supplied by the Washington University Department of Veterinary Medicine. ( *macaca mulatta*, monkey S: 7 kg, monkey B: 8 kg) participated in this study. Animals were housed in pairs with 12/12 hour light/dark cycles ^26^. Monkeys were fed on Purina Monkey Chow, fruit and treats, and were provided with environmental enrichment ^27^. We trained two male rhesus monkeys (macaca mulatta, 7 kg and 8 kg) to choose one of two targets using a saccadic eye movement or a reaching arm movement ^28^. Tests were performed during normal working hours (9am to 5pm). The animals sat head-fixed in a custom designed monkey chair (Crist Instrument) in a completely dark room. Visual stimuli (squares of 2.3° by 2.3°) were back-projected by a CRT projector onto a custom touch panel positioned 25 cm in front of the animals’ eyes. Eye position was monitored by a scleral search coil system (CNC Engineering). All procedures conformed to the Guide for the Care and Use of Laboratory Animals and were approved by the Washington University Institutional Animal Care and Use Committee.

### Task

Animals performed a two-alternative forced choice task. They first fixated and put their hand on a central target. After 120 ms, two white targets appeared simultaneously to the left and right of fixation. Each target was associated with a reward, described below. At the same time, the central fixation point changed color to either red or blue, instructing the monkeys that either a saccade or a reach, respectively, would be required on this trial. After a variable delay interval (0.8 s to 1.6 s), the fixation point disappeared, cueing the monkey to execute a movement to one or the other target. The animals’ behavior was very similar for choices made using saccades and reaches, and we therefore did not distinguish between the two. If they failed to make the instructed movement to within 7° of visual angle from one of the two targets within 1.5 s of fixation offset, then the animal received no reward and the start of the next trial was delayed by 2 s. Otherwise, the next trial started immediately after the reward was delivered.

The reward associated with the two targets consisted of a primary reinforcer—a drop of water, delivered by the opening of a valve for a particular length of time—combined with a secondary reinforcer—an auditory tone of the same duration. The volume of fluid delivered was proportional to the valve opening times. Our aim in designing the task was that at any one time, one target would deliver larger rewards than the other. The assignment of the richer and poorer targets to the left and right choices would change periodically, but in a way that would not be obvious to the animal or easy to determine. To accomplish this, we made many aspects of the reward delivery stochastic. At any one time, the mean reward durations for the two targets had a ratio of either 3 : 1 or 1.5 : 1. This ratio was held constant for a block of 7–17 trials (exponentially distributed with a mean of 11 trials and truncated at 17) and then changed to either 1 : 3 or 1 : 1.5. We used an exponential distribution of reward ratio duration because an exponential distribution has a flat hazard rate, making it difficult for the animals to anticipate a transition. Indeed, animals showed no anticipation of a transition ( Figure 2A). Within each block, the time that the water valve was held open in each trial was itself not held constant, but instead was drawn from a truncated exponential distribution that ranged from 20 to 400 ms. Thus, the valve open time differed from trial to trial, with an overall mean that differed for each target and changed every 7–17 trials. The effect of the exponential distribution was to make small rewards more common than large rewards, relative to the mean. This mean differed for each target and depended on the reward ratio for that block. For a reward ratio of 1.5 : 1, the mean valve open times for the richer and poorer target were centered around 140 and 70 ms, respectively. For a ratio of 3 : 1, the mean times were centered around 250 and 35 ms, respectively. To randomize reward delivery even further, the actual valve open times were multiplied by a factor ranging from 0.8 to 1.2, and this factor was changed on average every 70 trials (exponential distribution truncated to between 50 and 100 trials). Monkey A was trained in this specific task for about 6 months, monkey B for about 4 months. The data collection took about 6 months in each animal.

The reward magnitude of the option that the monkeys did not choose was assigned exactly in the same way as that assigned to the chosen option, that is, they were drawn stochastically from changing distributions with a particular mean. Once generated, the reward magnitudes for the unchosen option were fixed throughout the investigation.

### Data

The data are available in a .mat format at http://www.neuralgate.org/download/matchingdata and by clicking the link provided below.

Raw task data In this file, ‘choice’ is a binary vector of the animals’ choices (0 for a leftward and 1 for a rightward choice), ‘rewards’ is a two-column vector of the reward magnitudes (the left (right) column represents the reward magnitudes for the leftward (rightward) choices), and ‘meanreward’ is a vector indicating the current reward ratio ^39^. Click here for additional data file. Copyright: © 2015 Kubanek J and Snyder LH 2015 Data associated with the article are available under the terms of the Creative Commons Zero "No rights reserved" data waiver (CC0 1.0 Public domain dedication).

### Models

We modeled the monkeys’ trial-to-trial behavior using a mechanistic model. The model is grounded in reinforcement learning, a framework whose various instantiations have been applied previously to successfully explain foraging behavior ^6, 25, 29– 31^.

The model ( Figure 3) first computes the value *V* of each option by weighing the past 3 rewards *r _i_* obtained from choosing each option:

$$V\text{​}=\text{​}\sum_{i=1}^{3}w_{i}r_{i}.$$

The first two weights ( *w*_1_, *w*_2_) are free parameters; the third weight is *w*_3_ = 1 – *w*_1_ – *w*_2_ such that ∑ _i_ *w _i_* = 1.

The option that was chosen is assigned a value *r*_1_ = *R*, where *R* is the reward obtained for choosing that option. The unchosen option is assigned a value *r*_1_ = *ρ*, where *ρ* is a free parameter.

The value of the two options ( *V*_right_ and *V*_left_) are compared and a choice of the rightward option is made with probability

$$P_{\text{right}}=\Psi \left(V_{\text{right}}-V_{\text{left}}\right)=\frac{1}{1+\exp\left(\beta (V_{\text{right}}-V_{\text{left}})\right)},\;\;\;\;\;(2)$$

where the parameter *β* controls the steepness of the sigmoid function (see Figure 10).

The four parameters *w*_1_, *w*_2_, *ρ*, and *β* were fitted to the monkeys’ behavior such as to maximize the log likelihood log *L* that the monkeys’ choices could be made by the model:

$$\log L=\sum_{t}\log\left(P_{\text{right}}(t)c(t)+(1-P_{\text{right}}(t))(1-c(t))\right)$$

where *P*_right_( *t*) is the model’s prediction of the probability of choosing the rightward option on trial *t*; *c*( *t*) = 1 for the monkeys’ rightward choice on trial *t* and 0 for his leftward choice. The maximization was performed by the Nelder-Mead simplex direct search algorithm implemented by the function fminsearch in Matlab (The Mathworks, Inc., Natick, MA, RRID:nlx_153890). The algorithm converged in all tested conditions, and onto the same solution when run repeatedly.

We further simplified this model by approximating the three weights *w _i_* with a geometric sequence with the common ratio *α* ( Figure 8). Given that ∑ _*i*_ *w _i_* = 1, we can write $w_{1}=\frac{1}{1+\alpha +\alpha^{2}}\text{,}$ *w*_2_ = *αw*_1_ and *w*_3_ = *αw*_2_. We then fit *α* to minimize the mean squared error between the approximated and the actual weights.

We tested a variety of other models, none of which offered a significantly better fit. The present model is well established in the reinforcement learning literature ^29^, has been successfully used previously ^6, 30, 31^, and is a generalization of many special cases we also tested (see Results for an example).

We also tested an extended model that featured a separate set of weights for the unchosen option. This extension did not significantly improve the fit to the animals’ behavior or the ability of the freely foraging model to harvest more reward.

We further tested an extended model which in the ( *V*_right_– *V*_left_) term of Equation 2 featured two additional bias terms that could model the monkeys’ possible biases in choices made using saccades and reaches. These extensions had only minimal impact on the results (see Results). We therefore used the original, simpler model.

## Results

**Figure 1.**
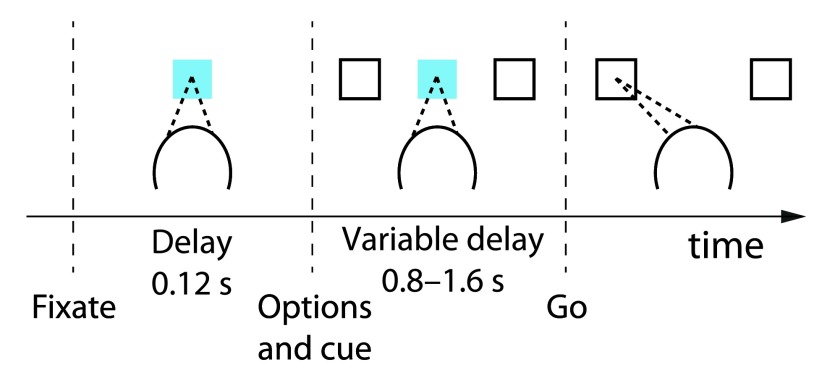
Foraging task with variable outcome magnitudes. Animals first fixated and put their hand on a central target. Following a short delay, two targets appeared in the periphery. The animals selected one of the targets using either an eye or hand movement, if the central cue was red or green, respectively. A choice was followed by the delivery of a liquid reward of a particular size. At any one time, one target was more valuable than the other, but individual rewards were stochastic and drawn from overlapping distributions, and which target was more valuable switched often and unpredictably. See text for details.

Monkeys engaged in a foraging task ( Figure 1) in which they selected one of two targets based on the associated reward magnitude. Specifically, one target was associated with a larger liquid reward than the other target, with mean payoff ratios of 1.5 : 1, 3 : 1, 1 : 1.5, or 1 : 3. The payoff ratio was held constant for 7–17 trials before changing to one of the opposite ratios. To further challenge the animals, the volume of juice delivered on each trial was variable, drawn from a truncated exponential distribution (see Methods for details).

**Figure 2.**
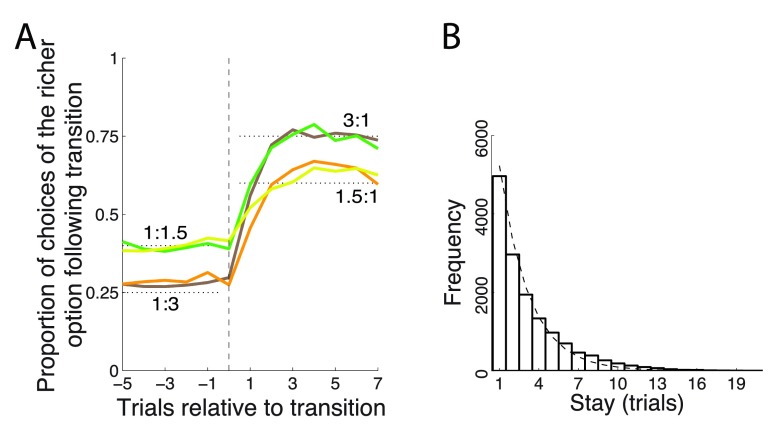
Matching behavior. ( **A**) Proportion of choices of an option as a function of each payoff ratio, aligned on a transition. The dotted black lines indicate the 3:1 and 1.5:1 proportions dictated by the matching law ( Equation 1). ( **B**) Frequency histogram of successive choices of one option. Dashed line: exponential fit.

The monkeys chose the richer option more frequently, but not stereotypically ( Figure 2A). On average, after each change of payoff ratio, the monkeys’ behavior converged in about 3 to 6 trials to a new steady state choice ratio. The fact that animals did not immediately switch over to a new steady state but required several trials to do so indicates that the animals were not aware of the transition times and integrated the reward history to converge onto the richer target. In the steady state (trial 7 following transition) the animals’ choices followed the strict matching law ( Equation 1). Specifically, for a ratio of 1.5 : 1, the strict matching law dictates choosing the richer option in 60% of trials. Our two animals chose the richer option in 60.0% and 61.6% of trials, respectively. For a ratio of 3 : 1, the matching law dictates choosing the richer option on 75% of trials. The animals chose this option in 73.5% and 71.9% of trials, respectively. Only the case of 71.9% slightly deviated from its corresponding matching level of 75% ( *p* = 0.022, *t*_1117_ = -2.29); the other three cases were indistinguishable from the corresponding matching levels ( *p* > 0.25).

The finding that animals matched the reward proportions in this task is notable given that we did not impose specific constraints typically used to elicit matching, such as reward baiting or change-over delay punishment of frequent switching ^1, 13, 23– 25^.

Animals switched from one target to another often ( Figure 2B), on average about once every third trial (probability to switch choice, *P* = 0.31). The distribution of stay durations was well approximated with an exponential ( Figure 2B), which suggests (though it does not prove) that the choice the animals made on a given trial was independent of the choice the animals made on the previous trial.

**Figure 3.**
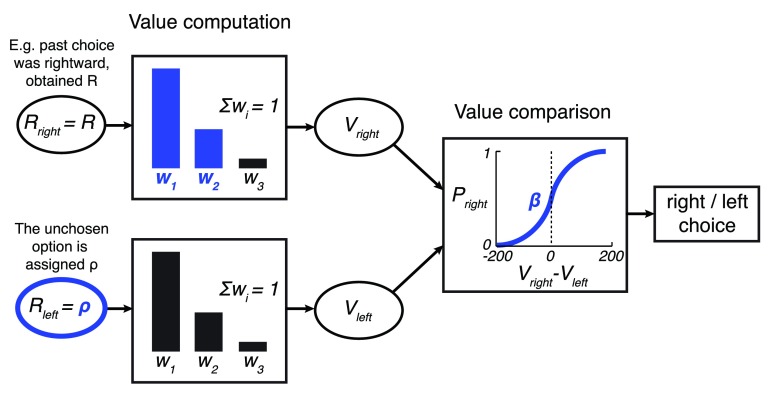
The model. In the model, a option is assigned the reward obtained from the according choice, of magnitude *R*. The unchosen option is assigned a value of *ρ*, a free parameter. The past three rewards obtained for each option *r _i_* are linearly weighted to obtain the value of an option, $V=\sum_{i=1}^{3}w_{i}r_{i}$. The weights *w*_1_ and *w*_2_ are free parameters; *w*_3_ = 1– *w*_1_– *w*_2_. The values *V*_right_ and *V*_left_ are then compared using a sigmoid choice function Ψ( *V*_right_ – *V*_left_) whose steepness is parametrized by *β*. This results in the model’s output: the probability of choosing the rightward option *P*_right_ in each trial. The model’s free parameters are highlighted in blue.

To gain insight into the processes leading to the matching behavior, we modeled the animals’ trial-to-trial behavior using a mechanistic model. The model (see Methods for details) is grounded in reinforcement learning and its various instantiations have been applied previously to successfully explain foraging behavior in reward-based tasks ^6, 25, 29– 31^. The model ( Figure 3) first computes the value *V* of each option. It does so by weighing the past three rewards *r _i_* obtained from choosing each option: $V=\sum_{i=1}^{3}w_{i}r_{i}.$ Two of the weights ( *w*_1_, *w*_2_) are free parameters; the third weight is *w*_3_ = 1 – *w*_1_ – *w*_2_ such that ∑ _*i*_ *w _i_* = 1. An important question is what reward magnitude the animals assign to the option that was not chosen. This reward magnitude constitutes an additional free parameter, *ρ*. Finally, the values of the two options, *V*_right_ and *V*_left_, are compared and a choice of the rightward option is made with probability *P*_right_ = Ψ( *V*_right_ – *V*_left_), where Ψ is a simple sigmoid function (see Methods, Equation 2) whose steepness is controlled by the parameter *β*. This sigmoid function can implement both a sharp *V*_right_ > *V*_left_) comparator function when *β* is large, as well as a more stochastic choice when *β* is small.

This framework is quite general and can represent many special cases. For instance, in a win-stay lose-shift (WSLS) model, an animal compares a just-obtained reward *R* against a threshold *T*; if *R* > *T*, the animal stays with its choice, else it shifts choice. This model is a special case of the above general framework in which free parameters *w*_1_ = 1, *w*_2_ = 0 (and so also *w*_3_ = 0), *ρ* = *T*, and *β* is large to achieve the sharp *R* > *T* comparator, e.g., *β* = 1.0.

We estimated the model’s four parameters such that the model’s predictions are close to the monkeys’ choices. The estimation was based on maximizing the likelihood of observing the monkeys’ choices given the model’s parameters (MLE; see Methods for details). The fit resulted in *w*_1_ = 0.816, *w*_2_ = 0.197 (and so *w*_3_ = -0.013), *ρ* = 55.1, and *β* = 0.023. We also tested an extended model by adding two additional parameters (one for choices made using saccades, one for choices made using reaches) at the comparator stage (see Methods for details) to account for possible biases in preferring a rightward or a leftward choice. This extended model resulted in very similar parameter fits ( *w*_1_ = 0.815, *w*_2_ = 0.198 (and so *w*_3_ = -0.013), *ρ* = 55.3, *β* = 0.023). Furthermore, the biasing values ( *V* = -4.6 and *V* = 8.5) were negligible compared to the large range of ( *V*_right_ – *V*_left_) (5th percentile equal to -172.8, 95th percentile equal to 176.3). We therefore used the simpler model.

**Figure 4.**
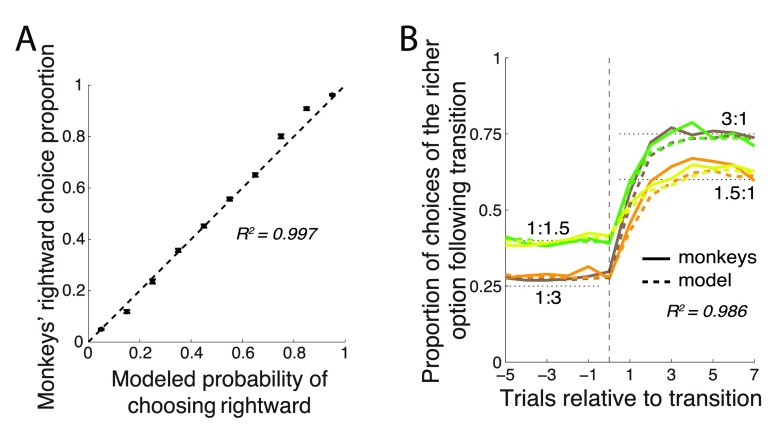
The model’s predictions. ( **A**) Proportion of choices of the rightward target (±SEM) as a function of the model’s probabilistic output, *P*_right_. ( **B**) Same format as in Figure 2A, with the model’s probabilistic output superimposed as dashed lines.

This simple model faithfully captured the animals’ behavior. When the animals’ choices were binned according to the model’s probabilistic predictions, there was a nearly linear ( *R* ^2^ = 0.997) relationship between the model’s predictions and the animals’ mean proportion of choices ( Figure 4A). For instance, across all trials in which the model claimed that *P*_right_ = 0.4, the monkey actually chose the rightward option in close to 40% of cases. The model also explained very faithfully the animals’ matching behavior and their behavior just after the payoff ratio transition ( Figure 4B). In particular, the model (dashed lines) explained *R* ^2^ = 0.986±0.005 (mean±SD) of the variance in the 4 curves.

When fitting the model, the model’s input (the rewards) and the outputs (choices) were held fixed; i.e., the model made the same choices as the monkeys and experienced the same rewards as the monkeys. Fixing the input and output permits us to investigate the structure of the model, i.e., to determine the mechanics of the transformation between the input and the output. However, it is also valuable to determine the model’s behavior, using the inferred parameters, when it is allowed to *make choices for itself*. This is important because it is conceivable that without the choice prescription, the model may show unstable behavior, such as alternating between choices or stereotypically making one choice.

**Figure 5.**
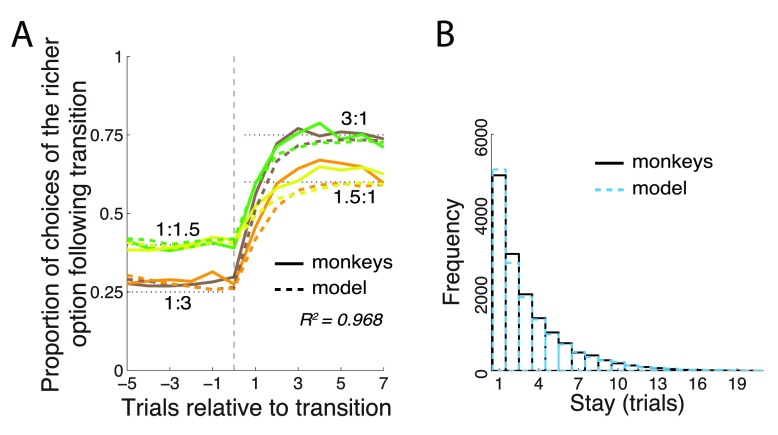
The model’s behavior when it made choices on its own. The model used the same parameter values as in Figure 4. Same format as in Figure 2, with the model’s behavior superimposed as dashed lines.

This was not the case. When the model made choices by itself (i.e., on every trial the model computed a *P*_right_ and made a rightward choice with probability *P*_right_), it still exhibited behavior similar to that of the monkeys ( Figure 5). Although the model chose the richer option slightly less frequently than the monkeys ( Figure 5A; 72.7% for 3:1 and 59.2% for 1.5:1), there was no significant difference between the monkeys’ and the model’s mean choice levels at the steady state for either the 3:1 or the 1.5:1 payoff ratios (trial 7 following transition, *p* > 0.11, t-tests). The model also exhibited trial-wise switch dynamics that were very similar to that of the monkeys ( Figure 5B). In particular, the mean stay duration of the monkeys (model) was 3.2 (3.3) trials; this small difference was not significant ( *p* = 0.19, *t*_28984_ = -1.31).

A question of particular interest is why the animals exhibited matching behavior in this task. We start this inquiry by asking whether the matching behavior was optimal in this task. An ideal agent who has information about the times of the payoff transitions will converge onto the richer option in one trial and continue to choose the richer option until the time of the next transition. Choosing the richer option at steady state in 100% of trials would constitute very strong over-matching. However, our subjects were not ideal: they were not signaled when the payoff transitions occurred, and we designed the task to make it difficult for them to detect the transition times. Specifically, the transitions occurred at random, exponentially distributed intervals, such that the hazard function for transition was flat. In addition, the reward magnitude received on each trial was variable, drawn from an exponential distribution (see Methods for details).

These task attributes may make it difficult for any subject or scheme to perform the task perfectly. To obtain an estimate of how well an agent might perform the task, we released the constraints on the model’s behavior and searched for the combination of parameter values that maximized the harvested reward. This reward-maximizing (“optimized”) model converges onto *w*_1_ = 0.621, *w*_2_ = 0.310 (and so *w*_3_ = 0.069), *ρ* = 72.4, and *β* = 0.207.

**Figure 6.**
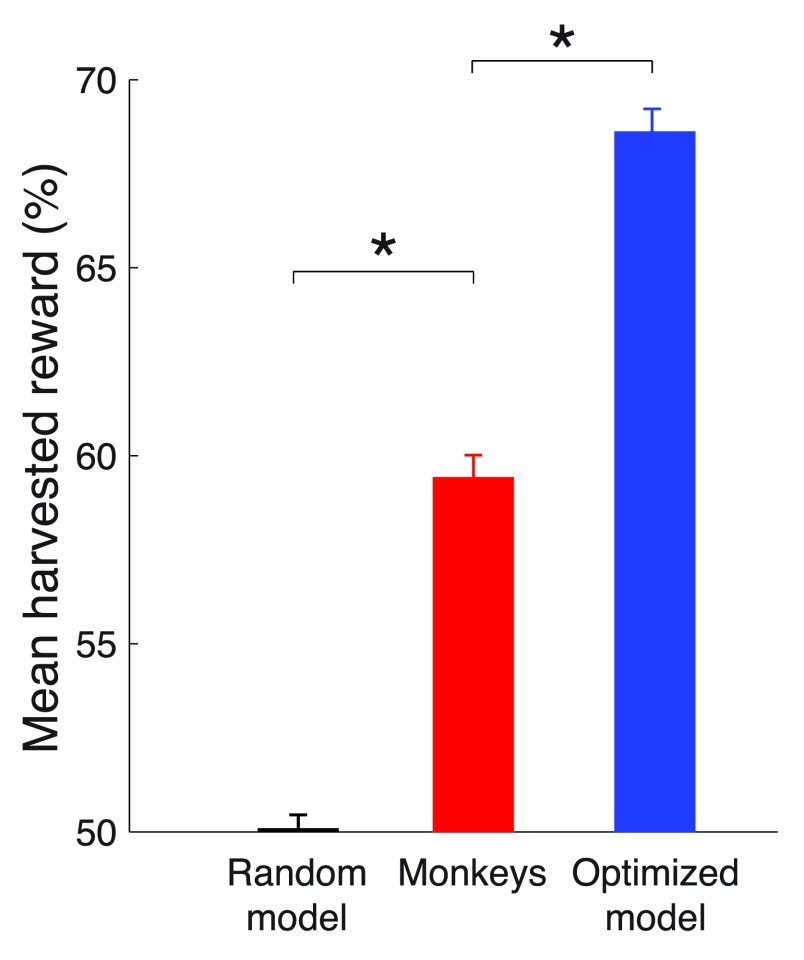
Comparison of mean harvested reward in the task. The mean reward harvested by a model that makes choices at random (defined as 50%), by the monkeys, and by the optimized model (see text for details). A theoretical maximum (100%) would be obtained by an ideal agent that has information about the payoff transitions times and always chooses the richer option. * *p* < 0.0001.

This optimized model harvested substantially more reward than the monkeys ( Figure 6). Choosing right and left options at random, which is equivalent to models that always choose the left or always choose the right option, will result in harvesting 105.9 ms of valve opening time per trial, which we label as random performance of 50%. The theoretical limit, achieved by an ideal agent that knows the transition times and so always selects the richer option, harvests 141.2 ms of valve open time per trial, which we label as 100%. Our moneys earned 59.4% of the reward on this scale. This was substantially more ( *p* < 0.0001, *t*_94306_ = 13.78) than the random choice model. However, the optimized model harvests 68.6% of the reward, substantially more ( *p* < 0.0001, *t*_94306_ = 10.99) than the monkeys. This result proves that the behavior of our monkeys was suboptimal in this task. Given the same reward environment, there is at least one physically realizable model that forages substantially better than the monkeys.

**Figure 7.**
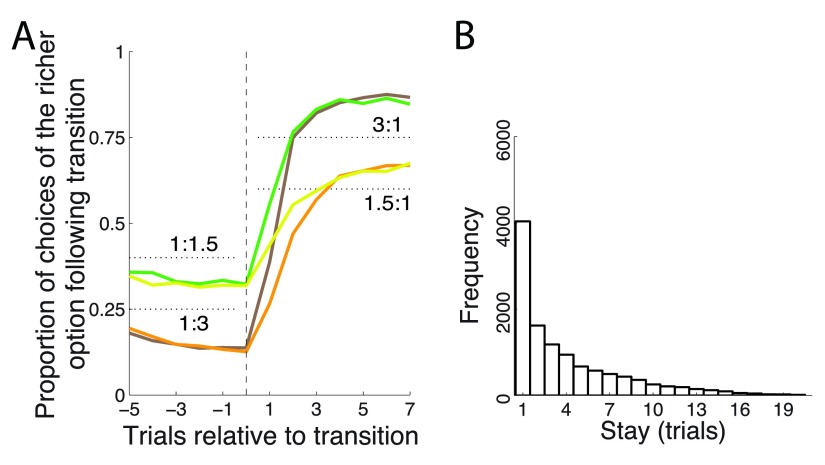
Behavior of the optimized model. Same format as in Figure 2, for the model with parameters maximizing its reward income.

The behavior of this optimized model is shown in Figure 7. As expected, the model clearly over-matches the reward proportions ( Figure 7A). The steady state proportions of choices of the richer option for the payoff ratios 3:1 and 1.5:1 were 85.7% and 67.2% respectively, both significantly different from the proportions dictated by the matching law ( *p* < 0.0001). The optimized model also switches less often than the monkeys ( Figure 7B), on average every 4.1 trials, compared to the 3.2 of the monkeys. The difference is significant ( *p* < 0.0001, *t*_26114_ = -20.83).

**Figure 8.**
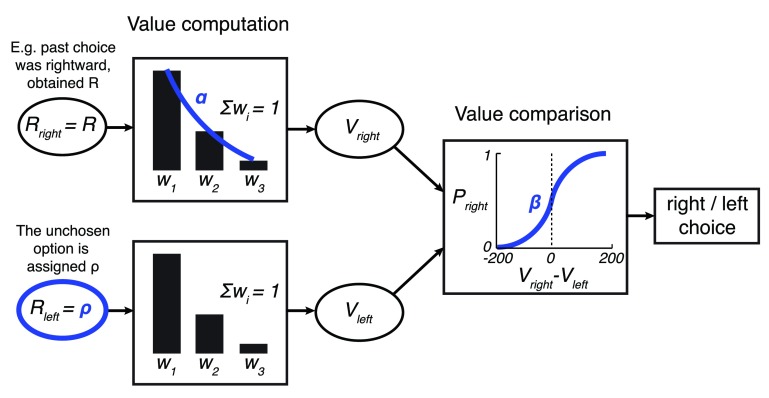
Simplified model. The model is identical to the model shown in Figure 3 with the exception that the weights are approximated with a geometric sequence with the common ratio *α*, subject to the constraint ∑ _*i*_ *w _i_* = 1. This way, $w_{1}=\frac{1}{1+\alpha +\alpha^{2}}\text{,}$ *w*_2_ = *αw*_1_ and *w*_3_ = *αw*_2_.

To simplify the presentation and interpretation of all that follows, we reduced the number of free parameters in the model from four to three ( Figure 8). A single parameter representing an exponential kernel replaces the two weight parameters ( *w*_1_ and *w*_2_). This is more biologically plausible than using multiple discrete weights. Note also that the weights of the monkeys’ data fit and the optimized model fit are well approximated by a geometric series, which is the effective result of an exponential kernel (monkeys: *w*_1_ = 0.815, *w*_2_ = 0.198, *w*_3_ = -0.013; model: *w*_1_ = 0.621, *w*_2_ = 0.310, *w*_3_ = 0.069). Taking into account the constraint ∑ _*i*_ *w _i_* = 1, the first weight *w*_1_ is approximated as $\frac{1}{1+\alpha +\alpha^{2}}\text{,}$ where *α* is the common ratio of the sequence. Then, *w*_2_ = *αw*_1_ and *w*_3_ = *αw*_2_. We set *α* such as to minimize the squared error between the actual weights and the approximated weights. That common ratio was found to be *α* = 0.201 for the model representing the monkeys, and *α* = 0.424 for the optimized model. The mean square error of these fits was small, equal to 0.058 for the model of the monkeys and 0.063 for the optimized foraging model. Consequently, the geometric approximation of the weights had negligible impact on the models’ behaviors (data not shown). The common ratio *α* helped not only to eliminate one free parameter; it also lends itself a straightforward interpretation: The larger the *α*, the more weight the monkeys put on the rewards received in the more distant past. For instance, for *α* =1, $w_{1}=w_{2}=w_{3}=\frac{1}{3}\text{.}$ Such model would simply average the past 3 rewards. The other extreme, *α* = 0 ( *w*_1_ = 1, *w*_2_ = *w*_3_ = 0) would only consider the last obtained reward. Henceforth, we refer to *α* as the model’s “memory”: The larger the *α*, the longer reward history is used to compute the value *V*.

We next investigated the role of the individual model parameters in the reward that can be harvested in this task. We visualized the effects of each parameter while fixing the values of the other two parameters. The fixed parameter values were the values of the optimized model ( *α* = 0.424, *ρ* = 72.4, *β* = 0.207), as this model is much closer to the optimum compared to the monkeys. The parameter *α* was varied between 0 and 1 in steps of 0.05; *ρ* between -100 and +300 in steps of 20; *β* from 10 ^-4^ to 10 ^2^ in geometric steps of 1.78. The parameter space additionally included also the values of the monkeys and of the optimal model.

**Figure 9.**
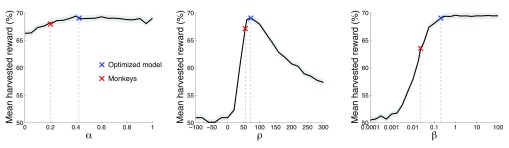
Reward as a function of the parameter values. Each plot shows the mean±SEM reward harvested as a function of a particular parameter value. We varied the value of a parameter while fixing the other two parameters at values of the optimized model ( *α* = 0.424, *ρ* = 72.4, *β* = 0.207). Red: parameters of the model of the monkeys’ behavior. Blue: parameters of the optimized model.

It is important to note that each two-dimensional plot of reward as a function of a parameter value only shows a slice through the reward landscape; it does not show the entire reward landscape, which for this three-parameter model is four-dimensional. Figure 9 shows the leverage of each parameters on the mean harvested reward given the fixed values of the other two parameters.

The model’s memory, *α*, had only small effect on the obtained reward. In regard to this aspect of the model, there was no significant difference ( *p* = 0.63, *t*_94306_ = -0.48) in the reward gained by the optimized model (blue) and the monkey model (red). Assuming that our model has mechanistic validity, this plot indicates that limits on memory, as captured by this parameter, are unlikely to underlie the monkeys’ suboptimal performance.

The reward assigned to the unchosen option, *ρ* (middle plot), had a strong leverage on the reward gained. There was a clear optimum centered around the value *ρ* ~ 70. The monkeys’ *ρ* = 55.1 fell somewhat short of the model’s *ρ* = 72.4. As a consequence, in regard to this parameter, the monkeys earned 2.9% less reward compared to the optimal model. Although this drop was significant ( *p* < 0.001, *t*_94306_ = -3.68), it can explain only about one-third of the monkeys’ suboptimal performance.

The parameter defining the steepness of the sigmoid that governs the value comparison ( Figure 8), *β*, strongly affects the reward that can be harvested (right plot). The monkey model and the optimized model differ substantially in the value of this parameter (monkeys: *β* = 0.023; model: *β* = 0.207). Compared to the optimized model which properly reached the optimum (within the convergence rules of the optimization procedure), the monkeys harvested 6.4% less reward than the model. This was a significant ( *p* < 0.0001, *t*_94306_ = -7.59) and substantial drop in the performance.

**Figure 10.**
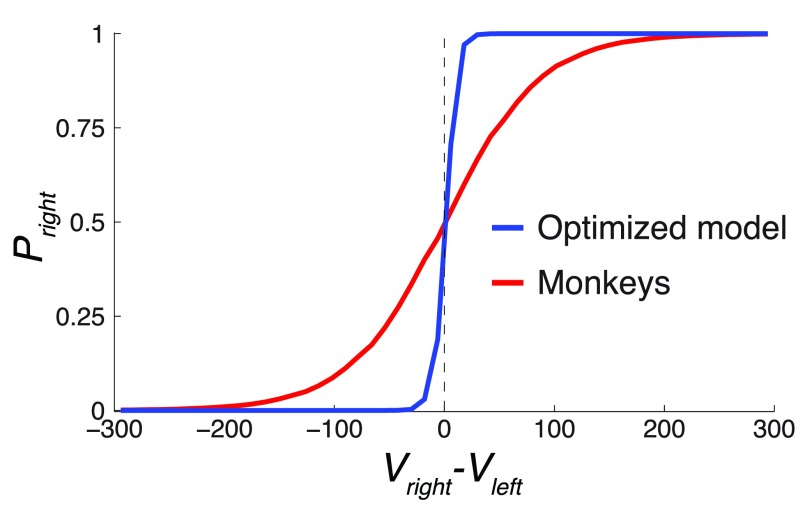
The value comparison function. The figure plots *P*_right_ = Ψ( *V*_right_– *V*_left_), over the range of ( *V*_right_– *V*_left_) (5th percentile equal to -172.8, 95th percentile equal to 176.3) for the Ψ parameter *β* of the monkeys and the optimized model. The optimized model had *β* about an order of magnitude higher than the monkeys, which defines its relatively sharp decision criterion.

Thus, the parameters *ρ* and *β* were instrumental in governing the gain in this task. Of these, the fit to the monkeys’ data suggests that their low value of *β* substantially impaired their performance. The effect of the relatively small value of *β* is plotted in Figure 10. The figure plots *P*_right_ = Ψ( *V*_right_ – *V*_left_), for the Ψ parameter *β* of the monkeys and the optimized model. The figure reveals that as a result of the relatively high *β*, the value comparison function of the optimized model is much steeper compared to that of the monkeys. As a result, the optimized model is better equipped to compare the two values when making a choice. In fact, the comparison function of the optimized model is so steep that it essentially acts as a perfect comparator, choosing the rightward option when *V*_right_ > *V*_left_ and the leftward option otherwise. The monkeys were not capable of performing such a sharp value comparison. As a result, their choice appeared more stochastic in regard to the value difference.

We next investigated why the monkeys did not achieve a steeper value comparison function given that its steepness *β* governs the amount of earned reward ( Figure 9-right). We hypothesized that this bottleneck may be due to the noisy representation of value (and value difference) by the monkey’s decision apparatus, which is presumably implemented by value-coding neurons ^32, 33^. The neuronal representation of value (and for that matter, of any variable) is inherently noisy ^34^. We simulated how well an ideal observer, given the spike counts of value-coding neurons, could distinguish *V*_right_ from *V*_left_. We will lay out an ideal case; as such, our estimate of the brain’s ability to distinguish the two values will likely be optimistic.

Neurons in many regions of the brain ^33, 35, 36^ increase their discharge rate ( *r*) with increasing value ( *V*) of the option they encode:

$$r=r_{\text{0}}+\theta V,\;(3)$$

where *r*_0_ is the baseline firing rate and *θ* is the slope of the linear relationship between firing rate and value. Thus, neurons that encode the value of the rightward option fire with rate *r*_right_ = *r*_0_+ *θ* *V*_right_ and neurons that encode the value of the leftward option fire with rate *r*_left_ = *r*_0_+ *θ* *V*_left_. We set *r*_0_ = 10 sp/s. We set *θ* to a 50% modulation of the baseline due to value, i.e., to *θ* = 5 sp/s over the value range (we used *V* = 300 as the maximum value).

Now, assume that an ideal observer, positioned as an idealized downstream decoder ^37^, knows which neurons encode *V*_right_ and which neurons encode *V*_left_. The task of this ideal observer is to tell, based on the discharge rates of these neurons *r*_right_ and *r*_left_, whether *V*_right_ > *V*_left_. For simplicity, we first consider the case in which the ideal observer assesses the activity of only one right-value-coding and one left-value-coding neuron. To be able to obtain any information from the spiking neurons, the ideal observer must measure the number of spikes *n* occurring within a certain time interval *T*. Because our monkeys had to make relatively fast decisions, we set *T* = 500 ms. Within this interval, the right-value-coding neuron will produce an average of *μ*_right_ = *r*_right_ *T* spikes; the left-value-coding neuron an average *μ*_left_ = *r*_left_ *T* spikes. These are average spike counts, however. Spikes occur stochastically; a different train of spike times will occur during each decision. We will model spike occurrence times using a homogenous Poisson process ^37^. As a result, during each decision, the measured spike counts *n*_right_ and *n*_left_ will be drawn from a Poisson (~ Gaussian for *n* > 10) distribution. The variance of these distributions is *σ* ^2^ = *μ*, i.e., $\sigma_{\text{right}}^{2}=r_{\text{right}}T$ and $\sigma_{\text{left}}^{2}=r_{\text{left}}T\text{.}$

**Figure 11.**
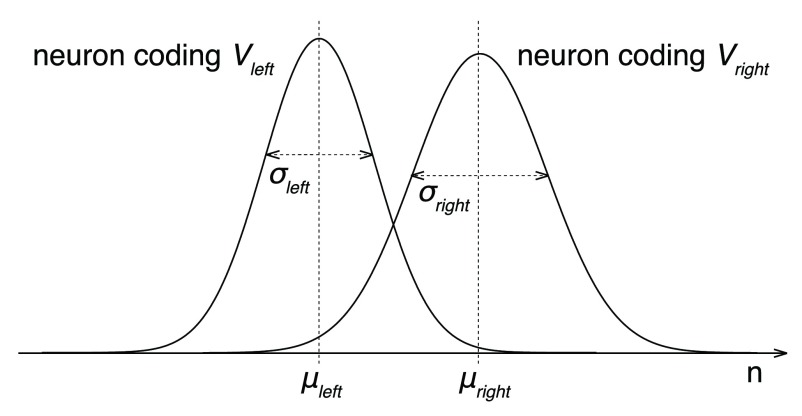
Representation of option values by spiking neurons. The plots show the distributions of spike counts *n* for a neuron encoding *V*_right_ and a neuron encoding *V*_left_. The spike counts follow a Poisson distribution. In the Poisson distribution, *σ* ^2^ = *μ*, so the right distribution with the higher *σ* also has a higher *α*. For large enough *n*, the distribution approaches a Gaussian. For simplicity, the illustrated distributions are Gaussian.

Due to the inherent noise in the spike generation process, the spike count distributions that encode the left and right value necessarily overlap ( Figure 11). As a consequence, even the ideal observer of neuronal spike counts will make erroneous judgments on whether *V*_right_ > *V*_left_. The probability of making a correct *V*_right_ > *V*_left_ decision Φ can be computed by drawing a boundary between the two distributions, and evaluating the rates of misclassification as a function of all boundary values (an ROC analysis ^37^). The area under the ROC curve then equals Φ. An alternative approach to evaluating Φ is to notice that comparison *V*_right_ > *V*_left_ is equivalent to *V*_right_ – *V*_left_ > 0. Thus, the ideal observer may simply evaluate whether *n*_diff_ = ( *n*_right_ – *n*_left_) > 0. Assuming that the two neurons fire spikes independently of each other, it is easy to show that the mean of *n*_diff_ equals *n*_right_– *n*_left_ and its variance equals $\sigma_{\text{right}}^{2}+\sigma_{\text{left}}^{2}\text{.}$ If *n*_right_ and *n*_left_ are close to normal, then their difference *n*_diff_ is, according to the central theorem, yet closer to normal. The resulting probability density function is *𝒩* $(n_{\text{right}}-n_{\text{left}},\sigma_{\text{right}}^{2}+\sigma_{\text{left}}^{2})=$ *𝒩* $\left(\frac{n_{\text{right}}-n_{\text{left}}}{\sigma_{\text{right}}^{2}+\sigma_{\text{lef}t}^{2}},1\right)\text{.}$ The probability Φ that *n*_diff_ > 0 then simply amounts to the integral below the normal probability density, which evaluates to $\text{erf}\left(\frac{n_{\text{right}}-n_{\text{left}}}{\sigma_{\text{right}}^{2}+\sigma_{\text{left}}^{2}}\right)\text{.}$ We are interested in the right tail ( *n*_diff_ > 0), so

$$\Phi =1-\text{erf}\left(\frac{n_{\text{right}}-n_{\text{left}}}{\sigma_{\text{right}}^{2}+\sigma_{\text{left}}^{2}}\right)=\text{erf}\left(\frac{n_{\text{left}}-n_{\text{right}}}{\sigma_{\text{right}}^{2}+\sigma_{\text{left}}^{2}}\right).\;\;\;\;\;(4)$$

(Note that $\left(\frac{n_{\text{left}}-n_{\text{right}}}{\sigma_{\text{right}}^{2}+\sigma_{\text{left}}^{2}}\right)\times 2=d'$, which is an often used measure of discriminability of two distributions in psychology and neuroscience.)

**Figure 12.**
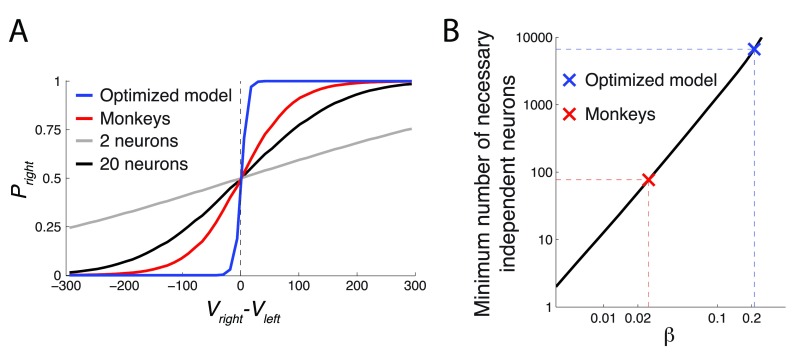
Increasing the steepness of the value comparison function costs an explosion in the number of required value-coding neurons. ( **A**) Same format as in Figure 10. The figure additionally includes responses of an ideal observer whose job is to tell *V*_right_ and *V*_left_ apart by reading out the responses of simulated spiking neurons (see text for details). The more independent neurons available to the ideal observer, the higher the ability to discriminate the two values. The gray (black) curve represent 2 (20) available neurons. ( **B**) The number of neurons necessary to obtain a value comparison function of a particular steepness ( *β*). The data are plotted in log-log space. In this space, the apparently linear relationship represents an exponential relationship between the two quantities. To increase *β*, one needs to access an exponentially higher number of independent value-coding neurons.

We presented the right-value-coding and the left-value-coding neuron with the range of values *V*_right_ and *V*_left_, respectively, experienced by the monkeys. Based on the spiking activity of these neurons, we plotted the probability Φ that the ideal observer could correctly choose the rightward option, i.e., *P*_right_ = Φ, as a function of *V*_right_ – *V*_left_ ( Figure 12A). The simple case of 2 independent neurons coding *V*_right_ and *V*_left_ is shown in gray. The plot reveals that the ideal observer can only poorly determine whether *V*_right_ or *V*_left_ is larger. There is too much noise in the spike counts.

The neuronal noise can be effectively reduced if the ideal observer can read out the activity of multiple uncorrelated neurons. In particular, if the observer averages the responses of *m* independently firing neurons in each (left or right) value-coding pool, then the noise variance *σ* ^2^ drops by a factor of *m*. As a result, the distributions of the average population spike counts become thinner than those of the individual neurons shown in Figure 11. Consequently, it is easier to tell the values drawn from these thinner distributions apart. Indeed, when the observer averages spike counts over 10 independent neurons in each pool (20 all together), the observer’s value assessment improves substantially (black curve in Figure 12A).

We plotted the minimum number of the independent value-coding neurons necessary to achieve the value comparison function of a particular value of *β*. The result is shown in Figure 12B. On the log-log scale plotted in the figure, there is an approximately linear relationship between the required number of neurons and the comparison function steepness *β*. This means that to achieve a higher *β*, one must employ an exponentially growing number of independent value-coding neurons. The minimum number of independent value-coding neurons to attain the *β* of the monkeys, in the ideal case, is 77. In contrast, the optimized model would require at least 6651 independent value-coding neurons.

It is important to stress that these numbers represent a theoretical minimum. We assumed neurons with a large (50%) modulation of their firing rates by value, assumed completely independent neurons (zero noise correlation), assumed that the ideal observer can flawlessly average the responses in the respective right and left neuronal populations, that the ideal observer has 500 ms of time to read out the spike counts during each decision, and disregarded any additional sources of noise. Therefore, the true numbers are likely to be substantially higher. Thus, this analysis suggests that increasing *β* to harvest more reward is very costly in terms of the number of neurons required. It is therefore likely that the neuronal noise presents a bottleneck in the animals’ attaining a steeper value comparison function.

**Figure 13.**
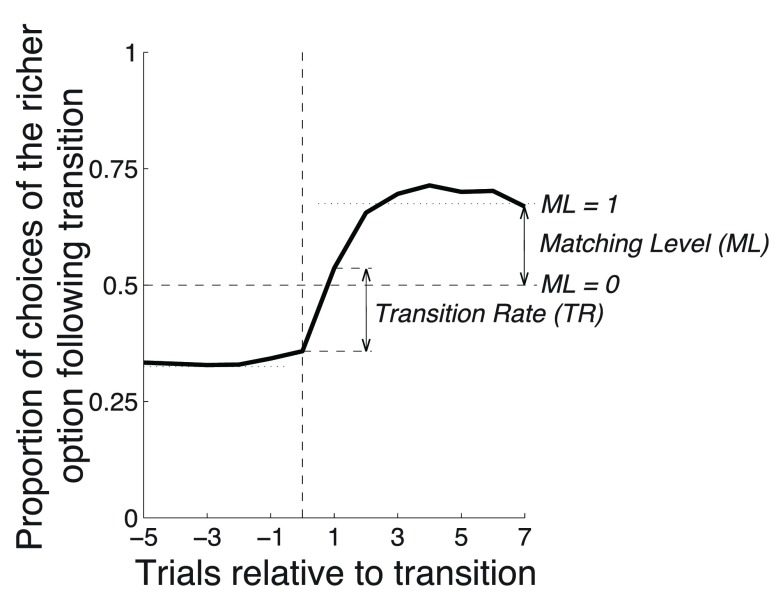
Transition Rate and Matching Level. The Transition Rate ( *TR*) is defined as the change in the proportion of choices of the richer option from trial 0 to trial 1 following transition. The matching level ( *ML*) is defined as the choice proportion at trial 7 following transition, such that *ML* = 0 for the 50% choice proportion and *ML* = 1 for the 67.5% proportion (average of 60% and 75%), with a linear continuum between and beyond these values.

Figure 7 revealed that the optimized model strongly overmatched the proportions dictated by the matching law. We next determined how the three model parameters of the simplified model influence two characteristics of the behavioral response: the matching level and the transition rate ( Figure 13). We define the matching level ( *ML*) as the choice proportion at trial 7 following a transition. We average across all four possible transitions (i.e., 1:3 reward ratio changing to 3:1 ratio, 1:3 ratio changing to 1.5:1 ratio, etc). We then scale the data such that selecting the two targets equally (unbiased or 50% choice proportion) corresponds to *ML* = 0, and perfect matching (average of 60% and 75%, or 67.5%) corresponds to *ML* = 1, with a linear continuum between and beyond these values. We define the transition rate ( *TR*) as the change in the proportion of choices of the richer option from trial 0 to trial 1 following transition, averaged across all four possible transitions.

**Figure 14.**
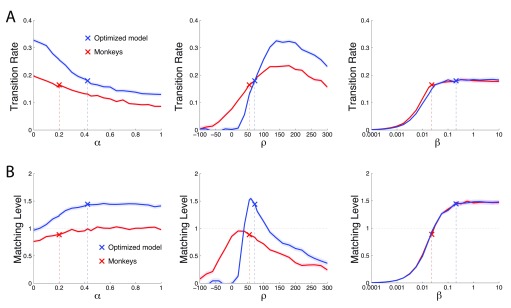
Transition Rate and Matching Level as a function of the parameter values. Same format as in Figure 9 but plotting Transition Rate ( **A**) and Matching Level ( **B**) instead of reward as the dependent variable. We varied the value of a parameter while fixing the other two parameters at values of the monkeys (red) and of the optimized model (blue).

We first evaluated the effects of each individual parameter on *TR* ( Figure 14A). The analysis is similar to that of Figure 9, except that the dependent variable is *TR* instead of reward. We evaluate the effect of each parameter on both the optimized model (blue: *α* = 0.424, *ρ* = 72.4, *β* = 0.207) and on the best-fit match to the monkey performance (red: *α* = 0.201, *ρ* = 55.1, and *β* = 0.023). The left panel reveals that *TR* is a monotonic function of the model’s memory *α*. As expected, the shorter the model’s reward memory (i.e., the smaller the reliance on the past rewards), the faster the model transitions to a new payoff ratio. *TR* is also strongly dependent on *ρ*, showing an optimum (middle panel). This is also as expected. During steady state, the poorer option is less often chosen. Therefore the larger the reward assigned to the unchosen option, the more likely that its value will exceed that of the chosen option, causing the model to switch. This benefit applies only up to a certain point: high values of *ρ* lead to metronome-like switching (not shown), thus hampering *TR*. *TR* is also sensitive to the steepness of the value comparator *β* (right panel). For a shallow comparator (low value of *β*), the model fails to clearly distinguish the values of the two options and as a result transitions poorly. This is improved by using a *β* of higher value, with an effect that saturates at just over *β* = 0.01.

In a similar vein, we then investigated which parameters are important in achieving a particular *ML*. To do so, we repeated the previous analysis, but for *ML* as the dependent variable ( Figure 14B). The model’s memory *α* has a small but noticeable effect on the *ML*. The longer the memory span (higher *α*), the higher the *ML*. This is as expected—reliably identifying the richer value requires a rigorous assessment of the past rewards; the weights on the past reward are maximal $\left(w_{1}=w_{2}=w_{3}\to \frac{1}{3}\right)$ when *α* → 1. The value of the reward of the unchosen option, *ρ*, has strong leverage on the *ML*. There is an optimum at about 0 < *ρ* < 80, depending on the values of the other two parameters. Notably, the *ρ* plot reveals that the optimized model did not maximize *ML*. Maximizing *ML* may not result in maximizing reward. We revisit this question at the end of the Results section. The steepness of the value comparison function, *β*, also had a substantial impact on the *ML*. The steeper the value comparison function, the higher the *ML*. This is as expected: the model should include as little noise in the value comparison as possible in order to correctly identify the richer option.

Finally, we investigated the possibility that animals optimized molar aspects of task performance, such as the *TR* and *ML*, instead of the parameters of the reinforcement learning model. We therefore plotted the mean harvested reward as a function of *TR* and *ML*. To obtain enough variability in these two attributes, we exhaustively tested each considered value of *α*, *ρ*, and *β* against each other. This resulted in 14283 different models, each associated with a *TR*, an *ML*, and a reward gain.

**Figure 15.**
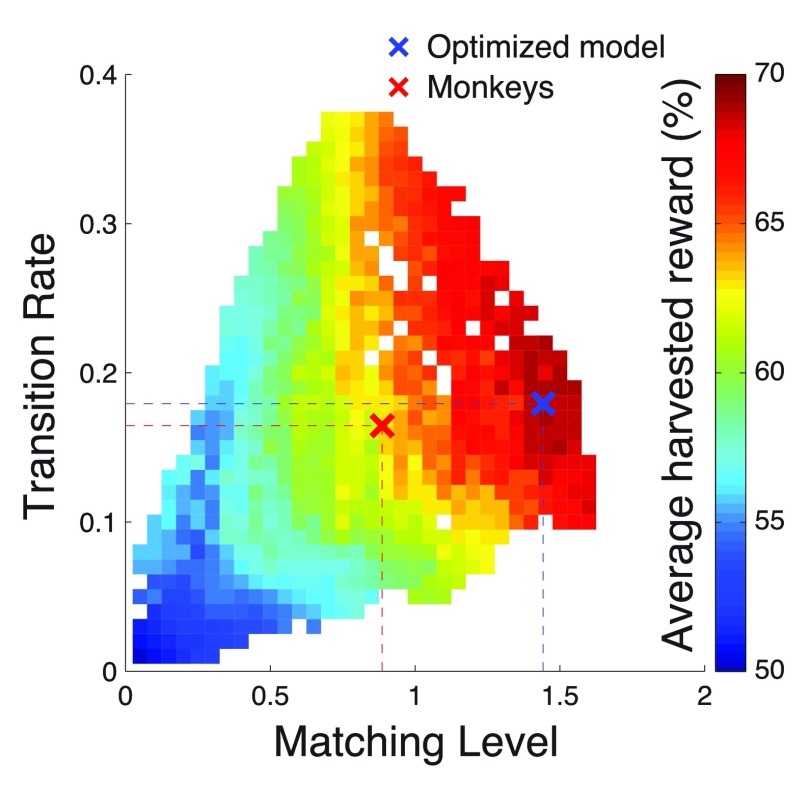
Average reward as a function of Transition Rate and Matching Level. We exhaustively varied, against each other, the values of *α*, *ρ*, and *β*, to arrive to a total of 14283 different models. Each was associated with a mean reward, with a Transition Rate, and with a Matching Level. The plot shows in color the mean harvested reward averaged over all models that have a particular value of Transition Rate and Matching Level. At the blank spaces, there was no model of the 14283 tested with the corresponding value of Transition Rate and Matching Level.

Figure 15 shows the mean harvested reward averaged over all models that have a particular value of *ML* and *TR*. The figure reveals that the mean reward increases both with increasing *ML* and increasing *TR*. This is as expected. An ideal agent should transition to the richer option as rapidly as possible, and in the steady state should maintain as high a value of *ML* as possible. Furthermore, the figure reveals that at certain level, there is tradeoff between *ML* and *TR*. In particular, starting at *ML* ≈ 1, a further increase in *ML* comes at the cost of a decrease in *TR*.

The model approximating the monkeys’ behavior (red cross) is positioned far from the maximum in this model-average reward landscape. There was no clear local optimum at that point, not in regard to *TR*, not in regard to *ML*, and not in regard to the particular combination of *TR* and *ML*. This suggests that the monkeys did not optimize their behavior based on *TR* or *ML*. The optimized model occupies a much more lucrative spot in this reward landscape, positioned at or near the maximum. Notably, the optimized model did not attain the highest value of *ML* it possibly could. Nonetheless, this allowed the model to achieve a higher *TR*. The plot shows that maximizing *ML* does not necessarily equal maximizing reward; it is important to strive for a high *TR*, too. However, at the high reward levels, there is a tradeoff between these two attributes of molar behavior.

## Discussion

Matching has been a widely studied and a much debated behavioral phenomenon ^1, 3– 12^. In baiting tasks, in which a reward, once scheduled, is available at an option until the subject harvests it, matching is the optimal or near-optimal strategy. In particular, it has been shown that matching follows from maximization of reward at either the molecular ^15– 17^ or molar ^13, 18, 19^ scales. Furthermore, at the level of mechanistic implementation, a biophysically based neural model grounded in reinforcement learning ^7^ was also shown to reproduce matching behavior in a baiting task ^25^.

An important question is to what extent matching applies to tasks that do not feature baiting or other control elements that render matching an optimal strategy. We engaged animals in a reward-based foraging task that featured neither baiting nor other controls to elicit matching. Surprisingly, we found that animals in our task very faithfully matched the reward proportions. This is a surprising finding because matching was not the optimal strategy in this task; we found that a model could harvest substantially more reward than the monkeys by over-matching the reward proportions. We investigated the source of the animals’ bottleneck at the mechanistic level. We found that the animals showed a relatively shallow comparison criterion that contrasts the values of the rightward and the leftward options ( Figure 10). This is an important bottleneck because at least in this task, the steepness of the value comparison function has a strong effect on the earned reward ( Figure 9, right). Furthermore, the steepness also has strong leverage on the level of matching ( Figure 14B, right).

One possible explanation for the animals’ poor comparison of the values of the options is that the they did not properly register the amount of the delivered juice. This is unlikely, for three reasons. First, there was a nearly linear relationship between the valve open time and the amount of fluid reward delivered (data not shown). Second, the setup produced an auditory beep of the duration corresponding to the valve open time, which served as a secondary reinforcer. A trained ear can likely distinguish duration differences of less than 5% ^38^. Third and most importantly, our pilot data showed that animals were capable of distinguishing even very small differences, namely a 105 ms from a 95 ms period of the valve opening.

If the suboptimal value comparison is not due to the registration of the reward magnitude, the bottleneck likely emerges from the internal representation of reward-related variables. There are many possible sources of noise affecting the representation of value in the brain. We considered the one that is inevitable and so at play: the noisy representation of value by spiking neurons. In a simulated representation of value by spiking neurons, we showed that the ability to discriminate two values is poor when only two neurons are considered in the discrimination ( Figure 12A). That ability improves when the number of independent value-coding neurons increases ( Figure 12A). Importantly, we found that the increase in the steepness of the value comparison *β* requires a recruitment of an exponential number of independent neurons ( Figure 12B). Thus, increasing the steepness of the value comparison function is very costly in regard to neural resources.

The finding that the animals’ value comparison function is relatively shallow indicates that the animals’ choice behavior is relatively stochastic. The simulation of the representation of value by noisy neurons provides one possible explanation for this stochastic choice behavior. However, the stochasticity might be also due to other factors. For instance, the animals might, at least in part, use a strategy that deviates from the optimal strategy of comparing the value of the two options. A deviation from that optimal strategy might appear as an increased level of noise in the animals’ choice. Another possibility is that the nervous system specifically introduces noise into certain stages of the decision machinery to promote foraging and exploration. This might be beneficial in environments with stochastic reward schedules, i.e., in which the reward obtainable for a choice is difficult to predict.

Notably, the statistical framework we employed in Figure 12 is general, not limited to the poisson noise in the spike counts. The analysis of the number of required neurons *n* simply rests on the fact that to reduce noise, one may average signals over *m* neurons; if the neurons are independent, the averaging reduces the variance in the noise by a factor of *m*. The simulation in Figure 12B showed that this rate of variance reduction is low with respect to an increase in the steepness of *β*: the relationship between *m* and *β* is exponential. Given this general statistical consideration, other forms of noise superimposed on the neuronal representations would lead to the same conclusion: To increase *β*, given a non-zero amount of noise in the brain, one must engage an exponentially growing number of neurons.

Conceivably, animals in this task could also under-match the reward proportions. However, under-matching would incur further loss ( Figure 15). In this task, matching thus appears as a compromise between harvesting a sufficient amount of reward and the demands placed by noise on optimal neural computation.

## Conclusions

We observed matching behavior in a task in which more reward could be harvested if animals over-matched the reward proportions. Mechanistic modeling revealed that the reward gained in this task and the level of matching strongly depend on the quality of the comparison of values of the decision options. The animals had a shallow comparison function, which dampened their reward income and their matching level. A neural simulation showed that an increase in the steepness of the comparison function is very costly (exponential explosion) in the number of the required value-coding neurons, given that there is a non-zero amount of noise in the neuronal representations. This finding identifies an important neural constraint on optimal choice.

## Data availability

The data referenced by this article are under copyright with the following copyright statement: Copyright: © 2015 Kubanek J and Snyder LH

Data associated with the article are available under the terms of the Creative Commons Zero "No rights reserved" data waiver (CC0 1.0 Public domain dedication). 

F1000Research: Dataset 1. Raw task data, 10.5256/f1000research.6574.d48853 ^39^
