# Supplementary material for: Matching Behavior as a Tradeoff Between Reward Maximization and Demands on Neural Computation
Source: F1000Res. 2015 Oct 2;4:147. Originally published 2015 Jun 9. [Version 2] doi: 10.12688/f1000research.6574.2 (PMC4654444; doi:10.12688/f1000research.6574.2)
Supplement: Peer review history

# Peer review: Referee response for version 2

**DOI:** 10.5256/f1000research.7688.r11179

**Published:** 2015-11-11

**Authors:** Jacqueline Gottlieb

**Affiliations:** 1 Department of Neuroscience, Columbia University, New York, NY, USA

The authors did a good job revising the paper, but they seem to have misunderstood my first comment about the block structure. My concern was not that the monkeys could have anticipated the block transitions (which I agree, the task design and data convincingly rule out). My question was whether, once they deduced that there had been a transition, the monkeys could have simply *switched* between the relatively few contexts they had over-learnt. Abrupt switching can produce a shallow change slope on average, if the switching happens at different points on different sessions. Given the weight that the authors put on a trial-by-trial learning mechanism, it seems important to consider and rule out alternative models.

I have read this submission. I believe that I have an appropriate level of expertise to confirm that it is of an acceptable scientific standard.

# Peer review: Referee response for version 1

**DOI:** 10.5256/f1000research.7059.r10361

**Published:** 2015-09-16

**Authors:** Jacqueline Gottlieb

**Affiliations:** 1 Department of Neuroscience, Columbia University, New York, NY, USA

This is a very interesting article that thoroughly examines the “matching” behaviour in monkeys using behavioural testing and reinforcement learning models. Monkeys perform a task where they can choose between two targets associated with variable rewards. The monkeys show approximate matching of the reward ratios in their choices, even though this is not optimal in the present task. The authors carry out an exhaustive modelling effort to characterize the matching behaviour, its difference from an optimized behaviour based on RL, and the parameters that give rise to non-optimality in the choices. Based on these efforts, they conclude that a significant source of non-optimality may be in the noise of internal value representations.

Overall the paper is very nicely done – it is well written and I greatly appreciate the thoroughness of the modelling efforts. I have several suggestions that may improve it:

 

1. By design, the authors provided the monkeys with reward magnitudes that varied in a complicated fashion in order to prevent stereotyped behaviors. However, above and beyond this variability, the *reward ratios* fell into only 4 distinct categories. Given enough training the monkeys could, in principle, have learnt these categories and used some stereotyped strategies to switch between them. The success of the RL- model in capturing the data seems to make this possibility unlikely – but this is not conclusive and there should be some explicit analysis of this possibility. At present there is no mention of the length of training (or even, in the data provided on the website, of the *session* from which a trial came from). These are important details to include.
2. Echoing the comment of reviewer 1, the conclusion that the source of suboptimality is in neural noise seems overdone. This is *one* possible explanation that lends itself to an elegant model, but the mapping function between behavior and neural activity is complex, and many other schemes are possible. The authors should discuss these alternative schemes.

I found the Introduction a bit difficult to follow. Although individual paragraphs are well written, I was not clear where the entire narrative was going. The analysis (in the Results) focuses on non-optimal choice strategies and their possible neural bases – and the Introduction should be re-arranged to bring out this theme.

I have read this submission. I believe that I have an appropriate level of expertise to confirm that it is of an acceptable scientific standard, however I have significant reservations, as outlined above.

# Peer review: Referee response for version 1

**DOI:** 10.5256/f1000research.7059.r10172

**Published:** 2015-09-01

**Authors:** Bruno Averbeck

**Affiliations:** 1 Laboratory of Neuropsychology, National Institutes of Health (NIH), Bethesda, MD, USA

The paper by Kubanek and Snyder presents interesting data and modeling on matching behavior. They find that animals match in a learning task where the animals should infer which of two targets will deliver the larger reward on each trial. They find matching behavior in the animals. The behavioral choice strategy of the animals is modeled using a simple value integration algorithm. The algorithm accounts well for the choices of the animals. They also show that the algorithm can significantly outperform the animals if it over-matches, i.e. if it picks the better option more often. The main difference between the improved algorithm and the animal's behavior is the decision noise or beta parameter. They then develop a second model which assumes that the noise in the animal's choice behavior is driven by limits in their population code for value. 

The paper is well written and the study has been carefully carried out. Overall, this is nice work. I would make one comment on the final conclusion, that the noisiness in the animal's choice behavior is driven by noise in their population code. Specifically, how can this hypothesis be differentiated from the possibility that the noise in the animal's choice behavior is a strategic choice? In other words, is the animal limited by noise in their population coding, or are they exploring for other reasons, including perhaps satisficing? Would their decision noise (the beta parameter) be the same in another task in which values have to be learned, but under different conditions?

I have read this submission. I believe that I have an appropriate level of expertise to confirm that it is of an acceptable scientific standard.
